# Navigating the Landscape of Cytometry-Based Single-Cell Proteomics: Quantification, Annotation, and Resources

**DOI:** 10.3390/ijms27083620

**Published:** 2026-04-18

**Authors:** Yangbo Dai, Ziqiang Liu, Bing Liu, Li Guo, Huaicheng Sun, Qingxia Yang

**Affiliations:** 1 State Key Laboratory for Organic Electronics and Information Displays, Institute of Advanced Materials (IAM), Nanjing University of Posts and Telecommunications, Nanjing 210023, China; 1023173017@njupt.edu.cn (Y.D.);; 2 Zhejiang Provincial Key Laboratory of Precision Diagnosis and Therapy for Major Gynecological Diseases, Women’s Hospital, Zhejiang University School of Medicine, Hangzhou 310058, China; 3 College of Pharmaceutical Sciences, Zhejiang University, Hangzhou 310058, China

**Keywords:** flow cytometry, single-cell proteomics, data processing, cell type annotation

## Abstract

Cytometry-based single-cell proteomics (CySCP) has emerged as a powerful tool for analyzing cellular heterogeneity at the protein level because of its ability to reveal dynamic cell states and response patterns through high-dimensional protein expression profiling in thousands of individual cells. However, detailed summaries of quantification, processing and analysis of CySCP data remain limited. This review provides comprehensive perspectives on CySCP, including quantification technologies, analysis pipelines, annotation strategies, and resource platforms. Specifically, first, the strengths and limitations of the detection platforms are discussed. Second, comprehensive data processing steps, including compensation, transformation, normalization, batch effect correction, signal cleaning, and doublets, debris or dead cells removal, are described in detail. Third, various strategies for cell type annotation, including manual gating, unsupervised clustering, supervised/semi-supervised classification, and fully automated approaches, are illustrated. Fourth, emerging CySCP databases, as critical resources for facilitating antibody validation, panel optimization, and open-access data sharing, are summarized. In summary, this review provides a comprehensive guide for the use of CySCP to obtain novel biological insights at the single-cell protein level.

## 1. Introduction

Single-cell proteomics (SCP) has emerged as an indispensable tool for analyzing cellular heterogeneity at the protein level [1,2]. As the most direct readout of cellular function, proteins capture signaling dynamics and phenotypic diversity that cannot be fully resolved by genomic or transcriptomic analyses. For decades, significant progress has been made in SCP techniques, such as mass spectrometry (MS)-based and cytometry-based approaches [3,4,5,6]. MS-based SCP enables unbiased identification and quantification of a wide array of proteins within individual cells, providing deep coverage of the proteome. Recent workflows routinely achieve the quantification of more than 5000 proteins per cell, with a standard single-day throughput of hundreds of cells; meanwhile, advanced automated and multiplexed platforms can boost throughput to over 1000 cells per day, while retaining unparalleled sensitivity for post-translational modifications, particularly in low-input and discovery-driven contexts [7,8,9,10,11,12]. In contrast, cytometry-based SCP (CySCP) has been established and widely adopted in both basic and translational research because of its scalability, standardized antibody panels, and compatibility with high-dimensional analysis. By enabling the simultaneous measurement of dozens of protein markers across tens of thousands of single cells in a single run, cytometry-based approaches have been particularly effective in characterizing cellular heterogeneity, dynamic states, and rare populations [13,14,15].

Recently, cytometry-based approaches have remained the most widely applied because of their scalability and compatibility [16]. In an SCP study based on cytometry, researchers typically perform data quantification [17], data processing [18], and data interpretation [19] to acquire biological insights. Data quantification corresponds to the measurement of protein expression using detection technologies and CySCP quantification techniques, such as conventional flow cytometry (CFC), spectral flow cytometry (SFC), and mass cytometry (MC) [20]. In data processing, the focus is on removing technical noise and batch effects to ensure accuracy and reproducibility, and compensation, transformation, normalization, and quality control are typically performed [21]. Data interpretation refers to downstream analyses of processed data, including cell type annotation and subsequent biological investigations. With respect to data interpretation, cell type annotation represents the most critical and, usually, the first step of single-cell downstream analysis. Annotation approaches include manual gating, unsupervised clustering, supervised/semi-supervised methods and fully automated tools [22].

Moreover, in addition to the wide range of computational methods developed for CySCP data analysis, a variety of data repositories and prior-knowledge resources have been established to support different stages of SCP projects, such as antibody panel design, marker selection, data sharing, and biological interpretation [23]. To the best of our knowledge, one or two aspects of CySCP analysis have been summarized in existing reviews, such as data processing or automated gating [24,25], but these reviews are now somewhat outdated in terms of both systems and methodologies. A systematic overview that integrates the key analytical aspects, along with the latest methods and resources, is still lacking, underscoring the need for a comprehensive framework in this field. Therefore, a systematic summary of the multiple analytical aspects of CySCP is needed.

**Figure 1:**
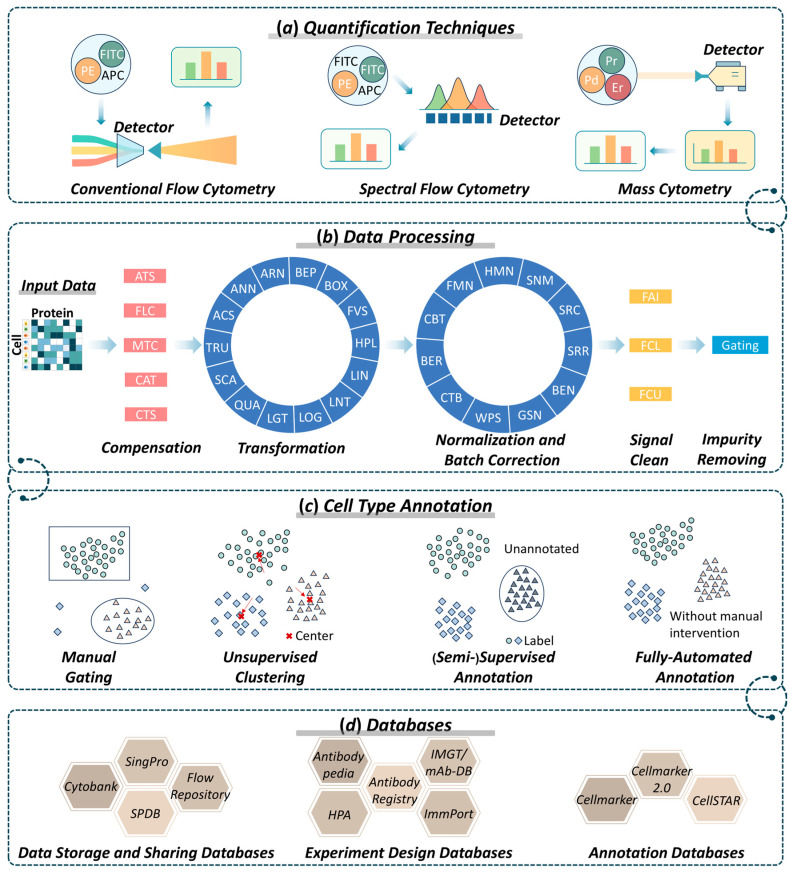
Flowchart of this review.

This review not only updates recent advances but also connects previously fragmented perspectives into an integrated analytical framework, connecting quantification technologies, computational workflows, cell-type annotation strategies and data resources (as shown in Figure 1). First, the fundamental principles of various techniques for CySCP are introduced, and their advantages, limitations, and applicable scenarios are highlighted. Second, a systematic data processing workflow for CySCP is summarized, which is accompanied by a thorough introduction to the specific methods corresponding to each step within the full data processing pipeline. Third, distinct strategies for cell-type annotation, ranging from manual gating to emerging automated annotation tools, are described. Fourth, the prevalent data resources for CySCP are introduced. Collectively, these sections provide a systematic overview of CySCP, highlighting the organization and coverage of current methodologies and resources. In summary, this integrative view aims to clarify how these layers collectively shape the analytical landscape of CySCP. This review might serve as a guide for the experimental design of CySCP and the processing and analysis of CySCP data to reveal novel biological insights at the single-cell protein level.

## 2. Cytometry-Based Quantification Techniques

Flow cytometry is a powerful technique for single-cell proteomics to characterize cellular phenotypes [26], identify biomarkers [27], and assess functional states [28]. Conventional flow cytometry is often constrained by issues such as fluorescence spillover and background autofluorescence, which limit its multiplexing capacity. To address these challenges, advanced quantification techniques, such as spectral flow cytometry [29] and mass cytometry [30], have emerged (as shown in Figure 1a), resulting in increased resolution or profiling depth. In parallel, MS-based SCP has achieved true proteome-scale depth and can routinely quantify several thousand proteins per cell with unparalleled sensitivity to post-translational modifications, but it continues to be restricted by the destructive nature of the measurement, substantially lower overall throughput, and the complete absence of live-cell sorting [31].

For applications requiring extreme scale, cell viability, and standardized translational panels, three cytometry-based platforms remain the unequivocal choice. As summarized in Table 1, conventional flow cytometry (CFC) is the most accessible and widely used method owing to its high throughput and live-cell compatibility. Spectral flow cytometry (SFC) extends multiplexing but requires complex unmixing. Mass cytometry (MC) offers high-dimensional, low-spillover measurements at the cost of throughput and viability, which uniquely enables unbiased proteome-wide depth but remains limited by destructive workflows and very low throughput. The following sections detail the principles, strengths, and current limitations of conventional flow cytometry, spectral flow cytometry, and mass cytometry, thereby providing the foundation for informed platform selection in contemporary single-cell proteomics studies.

## 3. Conventional Flow Cytometry

Conventional flow cytometry (CFC) is a laser-based methodology designed to measure the physical and biological characteristics of single cells (as shown in Figure 1a). CFC is among the most longstanding workhorses for analyzing cellular heterogeneity in single-cell studies [32,33]. In typical applications, antibodies can be designed according to the predefined cell population and experimental purpose, and an appropriate fluorochrome combination can be assigned to each selected marker via panel design [34,35]. For the antibody panel, the sample is stained with fluorochrome-conjugated antibodies. In the flow cytometer, when the cell passes through the laser area, each fluorophore is excited by the laser and emits photons of light with a unique spectrum [36]. Apart from the fluorescence signal, the CFC also measures the light scatter, including the forward scatter (FSC) measured in the forward direction and the side scatter (SSC) measured in the vertical direction. FSC and SSC are utilized for the discrimination of cells by size and granularity, respectively [37].

CFC has been used for single-cell analysis and cell sorting since 1965 [38]. The greatest advantage of CFC is its overwhelmingly high throughput (up to 100,000 cells per second) [39], which is critical for large-scale studies or rare subpopulation-relevant studies [40,41,42]. Furthermore, the widespread use of CFC is also attributed to other advantages, such as easy sample preparation and instrument operation protocols, low operating and maintenance costs, and the non-destructive nature of the whole process. Nevertheless, severe fluorescence spillover is a key limitation hindering the increase in resolution and multiplexing capabilities, making the development of high-parameter count panels in CFC extremely time-consuming and laborious [39]. Therefore, correcting fluorescence spillover is essential for CFC data analysis. This procedure, known as data compensation, can markedly improve the reliability of CFC results and reduce the difficulty of panel design. As a clinical application example, CFC has been successfully used to monitor measurable residual disease (MRD) in acute myeloid leukemia, achieving a sensitivity of 0.1% and demonstrating that MRD status strongly predicts relapse risk and overall survival [43]. This underscores how flow cytometry, despite its moderate multiplexing capacity, remains a cornerstone for rapid, quantitative single-cell proteomic assays in translational oncology. Overall, CFC offers unmatched throughput and accessibility, but its limited multiplexing ability constrains its role to applications where cell numbers and viability are prioritized over parameter depth.

## 4. Spectral Flow Cytometry

Spectral flow cytometry (SFC) is a rising technology and a transformative force in single-cell analysis [44]. SFC can be traced back to the initial phases of flow cytometry, and its use has become widespread because of advances in detector technology [29]. Compared with CFC, SFC uses a spectral detector array, which is typically composed of multiple photomultiplier tubes (PMTs) or avalanche photodiodes (APDs) arranged after a prism or diffraction grating, to capture the full emission spectrum of each fluorophore rather than only its peak (as shown in Figure 1a) [45]. In this way, SFC allows the design of larger panels with greater flexibility for fluorophores with similar emission peaks [46,47].

The most impressive feature of SFC is the ability to detect the full emission spectrum of each fluorophore, which markedly improves the multiplexing capability [39]. Although some SFC techniques can be used to analyze more parameters simultaneously, the availability of unique fluorochromes still limits the number of measurements per cell [48]. The powerful hardware platform of SFC promotes the development of novel fluorochromes with better and more distinct emission profiles [47]. Inheriting advantages from CFC, as a non-destructive technology, SFC enables the analysis or sorting of cells at a high rate (up to 40,000 cells per second). Unlike CFC, unmixing technology is used to distinguish the expression of different markers in SFC on the basis of reference controls. Moreover, during unmixing, cell autofluorescence can be extracted as a parameter and subtracted to significantly reduce the biological background noise in cells [46]. However, unmixing accuracy is highly dependent on the quality of single-stained reference controls, where dim or photobleached samples can introduce spreading error that rivals conventional compensation artifacts. These features make SFC particularly suitable for studies requiring greater multiplexing while retaining viable cells for downstream assays. A recent study leveraged full-spectrum flow cytometry to perform high-dimensional immunophenotyping of peripheral blood from common variable immunodeficiency (CVID) patients with COVID-19, identifying previously underappreciated CD21low naive B-cell subsets, exhausted CD4+ central memory T-cell populations, and dysregulated NK-cell differentiation states linked to persistent type I interferon signatures and inflammasome activation [49]. This example illustrates that spectral flow cytometry’s ability to unmix heavily overlapping fluorochrome signals, while preserving cell viability, supports discovery-oriented studies requiring both high parameter counts and functional cell recovery. However, its reliance on complex unmixing procedures and the limited availability of fluorochromes indicate that the full potential of SFC depends on advances in both computational tools and fluorochrome chemistry [50].

## 5. Mass Cytometry

Mass cytometry (MC), also referred to as cytometry by time-of-flight (CyTOF), is considered next-generation flow cytometry technology [51]. In 2009, metal isotopes were used to replace fluorophores and inductively coupled plasma time-of-flight mass spectrometry was applied for detection (as shown in Figure 1a), which created MC by combining the characteristics of flow cytometry and mass spectrometry [17]. MC soon became widely adopted in single-cell analysis because of its reduced signal spillover and ability to measure more parameters simultaneously compared with CFC [52,53]. For example, MC has been used to simultaneously quantify dozens of surface and intracellular markers, enabling the comprehensive characterization of diverse cell populations and their functional states [54].

The advantage of MC over other technologies is the high multiplexing capability (up to 60 parameters simultaneously) as well as the relatively low level of signal spillover between channels [55]. Although MC enables the measurement of more than 100 parameters in a single cell, most studies have detected 30–50 markers simultaneously because of the restrictions on appropriate metal-conjugated probes and signal spillover between different mass channels [56]. Notably, the signal spillover in MC is clearly less than that in CFC, which can result in some cell populations being falsely positive during cell type annotation and can complicate subsequent analysis [57,58]. Furthermore, another strength of MC is the rare biological background noise, because the metal isotopes used in MC are absent in human biospecimens [4]. However, MC has several limitations, such as low throughput and sample destruction upon analysis [59]. In CFC, non-toxic dyes enable both functional recovery and dead-cell exclusion. In mass cytometry, cells are vaporized during analysis, precluding any true viability measurement. However, viability gating remains feasible using metal-conjugated viability reagents that selectively label cells with compromised membranes prior to fixation. These signals allow computational removal of dead cells, ensuring that only intact cells contribute to downstream high-dimensional analyses. The throughput of the MC instrument is limited to 1000 cells per second because of the size of the single-cell-derived ion cloud, and a lower throughput is often adopted to reduce the number of doublet/multiplet events [60]. In addition, the destructive nature of the measurement prevents subsequent functional assays, and the requirement for specialized reagents increases costs. As a result, MC is particularly suited for deep phenotyping of limited samples rather than large-scale studies, and future developments in ionization efficiency and probe chemistry may help overcome the throughput and accessibility challenges. For instance, mass cytometry has been applied to map the immune landscape of solid tumors at single-cell resolution, revealing rare, therapy-resistant myeloid subpopulations and their spatial-like signaling networks that were missed by conventional flow cytometry [61]. This demonstrates that MC’s high-dimensional, low-spillover profiling is uniquely suited for dissecting complex tumor microenvironments and identifying novel targets for immunotherapy.

In summary, CFC prioritizes throughput and viability at the cost of multiplexing depth, SFC improves parameter capacity while retaining cell recovery, and MC maximizes phenotypic breadth but sacrifices speed and sample integrity. Selection among them ultimately reflects a trade-off between cell numbers, marker numbers, and the need for downstream functional assays.

## 6. Data Processing for Cytometry-Based SCP Data

**Figure 2:**
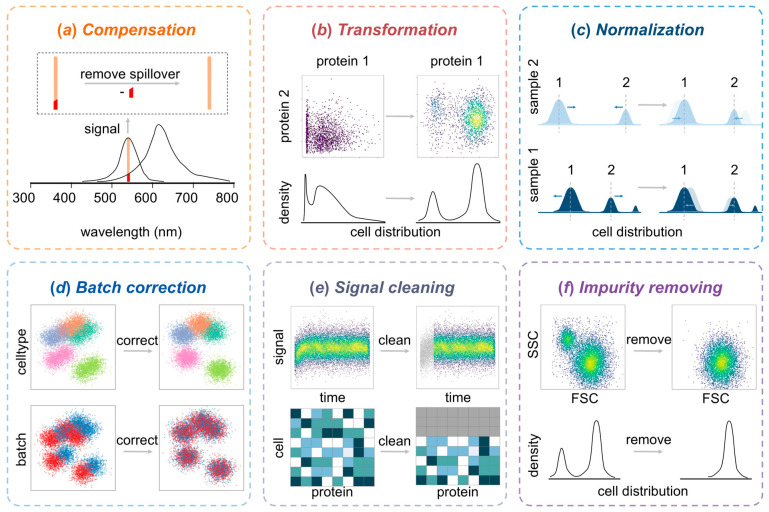
Data processing pipeline for cytometry-based single-cell proteomics. (**a**) Compensation corrects for signal spillover between fluorescent channels, thereby enhancing the accuracy of multiparameter detection. (**b**) Data transformation applies mathematical approaches to optimize the distribution of cell populations, aligning them with the assumptions of statistical models. (**c**,**d**) Data normalization and batch correction minimize technical variation across experimental batches or platforms, ensuring the consistency and comparability of the analytical results. (**e**) Signal cleaning filters out low-quality events caused by instrument malfunctions, improving the overall reliability of the dataset. (**f**) Single-cell gating uses physical parameters and viability markers to exclude doublets, debris, and dead cells and retain only biologically active single cells for downstream analysis. In subfigure (**b**–**f**), the applied color gradient indicates cellular event density: warmer and brighter colors (yellow-green) represent regions with higher cell abundance and denser cell clusters, while cooler, darker colors correspond to sparser cell distributions or background noise/debris. In subfigure (**c**), different blue shades distinguish Sample 1 (dark blue) and Sample 2 (light blue). Labels 1 and 2 denote two separate cellular subpopulation signal peaks. Arrows demonstrate the normalization step, which aligns these peak distributions across samples. In subfigure (**d**), distinct discrete colors are used to separately denote unique predefined cell types as well as distinct experimental batches before and after batch correction.

Because of the complexity of CySCP data, appropriately processed high-quality data are needed to ensure the accuracy and robustness of the results [62]. Proper data processing is critical because of the existing technical variations, signal noise, and inappropriate data distribution [63]. A structured workflow typically consists of compensation, transformation, normalization, batch correction, signal cleaning, and the removal of doublets, debris, and dead cells, as summarized in Figure 2 [64,65,66,67,68,69]. Notably, each component of the workflow targets a specific source of low-quality signal or aims to optimize the data distribution, and not all steps are universally needed.

Large-scale benchmarking indicates that processing choices profoundly influence downstream biological interpretation. ANPELA 2.0 [21] was used to evaluate 1125 pipelines across multiple reference datasets and demonstrate that inappropriate compensation, transformation, or batch correction significantly degrades clustering accuracy and introduces artifacts in pseudo-time inference. These results indicate that processing methods have a major effect on the reliability of downstream biological interpretation in CySCP. In the following sections, the rationale, representative methods, and key considerations for each step are outlined to provide guidance on selecting the most appropriate strategies to achieve robust insights. Representative methods and tools for each step are briefly listed in Table 2.

## 7. Compensation

Compensation corrects unwanted signal spillover to reflect the true contribution of each fluorochrome or metal isotope-conjugated probe (as shown in Figure 2a) [70]. With respect to CFC, traditional matrix-based compensation remains mainstream when instrument software such as the FlowCore v2.22.1 R package [72], which uses single-stain controls to estimate spillover matrices before inversion for correction, is used [90]. AutoSpill has recently improved reproducibility in CFC compensation by iteratively refining spillover matrices through robust linear models and reducing reliance on manual intervention [70].

With respect to SFC data, unmixing is a good substitute for compensation to distinguish the spectra of different fluorochromes on the basis of single-color controls and removing autofluorescence on the basis of unstained controls [48]. In fact, most studies use the software provided by instrument suppliers to unmix SFC data in real-time or post-acquisition [91]. For MC data, spillover is generated from isotopic impurities, oxide formation, and abundance sensitivity rather than from interference between channels [57]. Spillover is also an existing signal artifact that should be corrected, although the impact of spillover in MC is obviously less than that in CFC [58]. A compensation method for MC, the CATALYST R package, was proposed to generate a spillover matrix using staining polystyrene antibody-capture beads, estimating spillover and computing the compensation matrix using either conventional least-squares or non-negative least-squares regression [58]. Recently, CytoSpill was presented as an alternative to correct the spill effects in MC data without single-stained controls [71].

In practice, the applicability of compensation methods is often constrained by the availability of auxiliary data. Empirical analysis across multiple benchmarks reveals that methods requiring additional spillover information are frequently inapplicable in public datasets due to the absence of single-stained controls. In contrast, methods such as CytoSpill, which do not require additional inputs, exhibit greater practical utility, although their performance remains highly dataset-dependent.

Together, these approaches highlight how compensation strategies are closely tied to platform-specific characteristics. While CFC is mainly challenged by fluorescence spillover, SFC must address spectral overlap and autofluorescence, and MC correction focuses on isotopic impurities. Recent algorithmic advances such as AutoSpill and CytoSpill reduce reliance on manual intervention and improve reproducibility, but their accuracy still depends on experimental design and control quality. Future work may need to further integrate experimental and computational solutions to achieve more robust compensation across increasingly complex and high-dimensional panels.

## 8. Data Transformation

Data transformation is a vital processing step in which the raw CySCP data are converted into more symmetric normal distributions and the heteroscedasticity of the raw data is corrected (as shown in Figure 2b) [80,92]. Proper data transformation methods can distinguish cell populations and improve the performance of cell type annotation [92,93]. Logarithmic transformation is typically used [85], although alternatives such as arcsinh transformation [74,94,95,96,97,98,99], Logicle transformation [81], and FlowVS transformation [77] are employed when negative values or background noise are problematic. Additionally, detailed descriptions of more representative and novel data transformation methods are introduced in Table 2.

Recent benchmarking studies have highlighted significant performance disparities between transformation methods. Arcsinh transformation consistently ranks among the top-performing workflows for both cell subpopulation identification (CSI) and pseudo-time trajectory inference (PTI) tasks, reinforcing its status as the default setting in established platforms like PhenoGraph. Conversely, Quantile transformation is frequently associated with low-performing workflows and has been seldom recommended in recent CySCP literature.

Overall, transformation methods not only improve data symmetry and stability but also directly impact downstream analyses such as clustering and cell type annotation. A remaining challenge is balancing flexibility with reproducibility, since parameter tuning can introduce operator bias. Establishing standardized guidelines or automated parameter selection may help ensure that transformation enhances comparability across studies without compromising the detection of rare but biologically meaningful populations.

## 9. Data Normalization and Batch Effect Correction

Data normalization and batch effect correction can reduce the technical variation across different instrumentations, operators, and batches to improve the comparability of CySCP data (as shown in Figure 2c,d) [100]. For CFC, methods such as GaussNorm and warpSet normalize data on the basis of landmark identification and kernel density estimation [83,84]. However, SFC lacks standardized normalization methods, and few studies normalize the dataset before data analysis. For MC data, the bead-based normalization method has been widely employed and has become a standard processing step to correct signal variability because of changes in instrument performance [101,102,103,104,105]. Moreover, computational tools, such as CytofBatchAdjust (available at https://github.com/CUHIMSR/CytofBatchAdjust, accessed on 17 April 2026) [87] and BatchEffectRemoval (available at https://github.com/ushaham/batchEffectRemoval2020, accessed on 17 April 2026) [85], have been developed to further remove batch effects for MC data. More details on the normalization and batch effect correction methods are provided in Table 2.

The effectiveness of normalization is intrinsically task-dependent. For instance, Min-Max Normalization tends to outperform other methods in CSI tasks, whereas Gaussian Normalization shows superior performance in PTI studies. This variance underscores the importance of selecting normalization strategies that align with the specific biological objectives of the analysis.

Taken together, normalization and batch effect correction remain a critical bottleneck in ensuring data comparability across studies. While bead-based normalization has become routine for MC, CFC and SFC still lack standardized, universally adopted solutions. The increasing application of machine learning models such as BatchEffectRemoval illustrates a promising direction, but their “black box” nature can reduce interpretability. Thus, future work may need to integrate both statistical rigor and biological validation to guarantee that batch correction preserves true biological signals without overfitting to technical noise.

## 10. Signal Cleaning

Signal cleaning is used to remove the erroneous events resulting from abnormal interference from the instrument, such as bubbles, large particulates, and speed changes (as shown in Figure 2e) [106]. Manual removal of erroneous events is difficult and time-consuming, so several methods (such as flowClean [88], flowAI [33], flowCut [89], and PeacoQC [107]) have been proposed to identify and remove erroneous events automatically prior to downstream analysis. Specifically, flowClean groups cells into populations and applies changepoint analysis to determine the abnormal events without removing them [88]. flowAI can remove erroneous events from abrupt speed changes, unstable signal acquisition, and outliers of the dynamic range [33]. flowCut identifies erroneous events on the basis of the density of events and eight statistical measures [89]. PeacoQC removes the peaks with erroneous events based on the isolation tree method and the mean absolute deviation distances. PeacoQC is more applicable for SFC and MC data, and flowClean, flowAI, and flowCut are designed for CFC data [107]. Moreover, detailed descriptions of the signal cleaning methods are shown in Table 2.

While advanced signal cleaning tools like PeacoQC v1.20.0 are designed to enhance data quality, they do not always yield superior results across all datasets. Empirical evaluations indicate that in certain CSI benchmarks, omitting additional cleaning can lead to more stable outcomes, whereas tools like FlowAI v1.40.0 demonstrate advantages in PTI tasks. This highlights the technical trade-offs between algorithmic complexity and actual denoising efficacy in real-world scenarios.

Although these tools have improved automation in data preprocessing, signal cleaning remains highly context-dependent. Overly aggressive filtering can lead to the loss of rare but biologically relevant events, whereas insufficient cleaning may introduce artifacts into downstream analyses. Current methods are mainly reactive and identify artifacts after acquisition; however, preventive strategies such as real-time monitoring during acquisition or hybrid approaches that combine instrument-level quality controls with computational filtering could further enhance data reliability.

## 11. Removal of Doublets, Debris and Dead Cells

Doublets, debris and dead cells are excluded from the analysis to ensure the accuracy of cell type annotation and other subsequent analyses [108,109]. For CFC and SFC data, doublets are typically removed by gating cells with similar heights or widths but larger areas or by using sequential gates (as shown in Figure 2f) [110,111]. Debris is gated using FSC and SSC, but overlap with cell populations can be an issue [112,113]. Therefore, operations (such as Ficoll/Hypaque density gradient) are recommended during sample preparation for samples with prevalent debris [114]. Moreover, dead cells in CFC and SFC can be gated using a channel of viability dye such as DAPI [115].

With respect to MC data, doublets and other multiplets can be removed using the event length and a channel of DNA intercalators [116]. Some instruments (such as the Helios mass cytometer) can store several Gaussian parameters in the FCS file to remove doublets by the event length and residual [117]. These strategies are implemented in platforms or R packages, such as Cytobank [118], FlowJo [119], flowCore [72], flowStats [83,109], and OpenCyto [120].

Despite the availability of these strategies, doublet and debris removal continues to face challenges in terms of balancing sensitivity and specificity. Conservative gating risks retaining unwanted artifacts, whereas stringent thresholds may lead to the loss of meaningful rare populations. Furthermore, differences between platforms (CFC, SFC, and MC) complicate the adoption of standardized criteria. Future developments may benefit from machine learning-based approaches that integrate multiple parameters simultaneously, offering more nuanced discrimination while minimizing subjective bias in gating.

Overall, these empirical findings reveal that there is no ‘one-size-fits-all’ pipeline for CySCP data. The inherent variability in method performance across different benchmarks emphasizes the necessity for adaptive, task-specific workflow recommendations to ensure the stability and reproducibility of single-cell proteomic markers.

## 12. Cell Type Annotation of Cytometry-Based SCP

**Figure 3:**
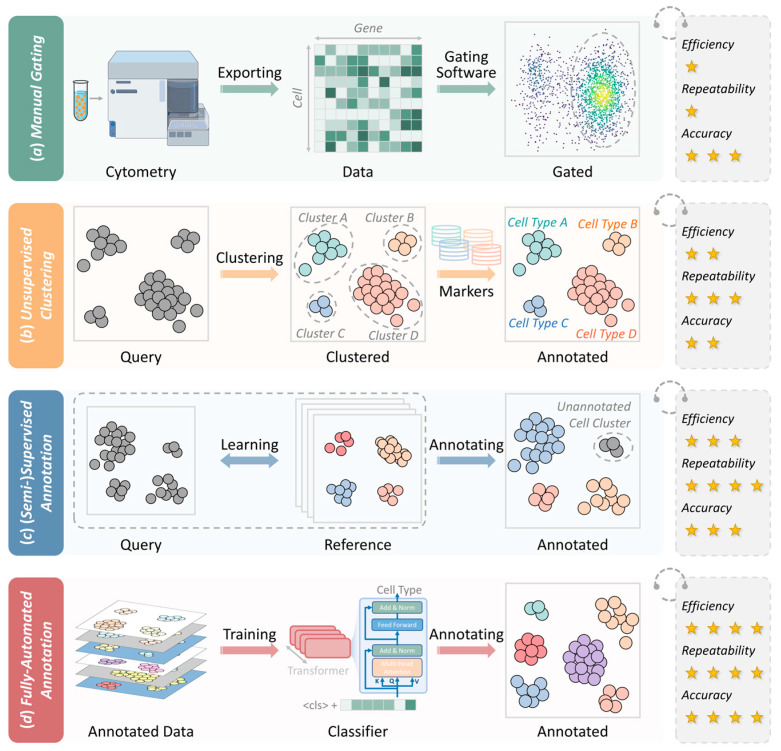
Four strategies for cell-type annotation. (**a**) Manual gating: Manual delineation of target cell populations on two-dimensional scatter plots using polygons or ellipses; this approach relies heavily on operator expertise and is generally low in efficiency. (**b**) Unsupervised clustering: Algorithms such as FlowSOM automatically cluster cells and subsequently perform dimensionality reduction to visualize subpopulation distributions and achieve marker-based biological annotation. (**c**) Supervised/semi-supervised annotation: Labeled data train classification models (such as RF or SVM), with the performance depending on the representativeness of the training data. (**d**) Fully automated annotation: Models built on large, high-quality reference datasets use machine learning for automated feature selection and efficient annotation. The number of stars (★) indicates relative performance; more stars represent better efficiency, repeatability, or accuracy. In the schematic diagrams, gray clusters denote unannotated cells, and multi-colored clusters correspond to different biologically annotated cell types.

Cell type annotation is the most critical step in the analysis of SCP data and refers to the identification of the cell types or subtypes present in a given experiment. Cell type annotation forms the foundation of downstream analyses by determining the identity of each cell and enabling applications such as cell population quantification, biomarker discovery, and functional characterization. As shown in Figure 3, annotation strategies encompass multiple approaches, such as traditional manual gating and advanced automated methods. In early studies with limited cell numbers and fewer measured proteins, annotation was typically performed manually through gating. However, with the rapid increase in both the dataset size and number of detectable proteins, manual gating has become labor-intensive, time-consuming, and subject to operator bias. Consequently, it has been increasingly replaced by automated or semi-automated approaches based on statistical modeling and artificial intelligence (AI). The transition from manual to automated methods is not merely a shift in efficiency, but a fundamental change in how the field addresses experimental reproducibility and algorithmic bias. Despite the proliferation of tools, the lack of standardized benchmarking remains a significant hurdle for cross-study comparisons. The evolution of these annotation strategies, along with representative methods, is discussed in detail below to assist CySCP researchers in achieving efficient and accurate cell type annotation.

## 13. Manual Gating for Cell Type Annotation

In the early stages, conventional flow cytometry supported only 4–6 signal channels. Researchers have relied primarily on expert knowledge to manually gate cells using two-dimensional scatter plots to accurately annotate cell types (as shown in Figure 3a) [121]. Because of its interpretability and visual intuitiveness, the manual gating approach is still considered the gold standard for cell type annotation [122,123].

However, with the growing adoption of mass cytometry and single-cell multi-omics, the dimensionality and complexity of CySCP data have increased exponentially. For instance, mass cytometry can measure more than 50 markers simultaneously and analyze more than a million cells in a single study [124]. Given the complexity of such high-dimensional data, relying solely on manual gating is a time- and labor-intensive process with limited reproducibility [125]. The inherent subjectivity of this approach—often termed “operator bias”—is a significant limitation, as already evident in lower-dimensional fluorescence flow cytometry. Even highly trained experts can produce divergent results when analyzing the same dataset, because the boundary between positive and negative expression is often arbitrary. These discrepancies are exacerbated in high-dimensional CyTOF data, where exhaustive visualization of all marker combinations is impossible. This lack of a formal benchmarking standard makes it difficult to replicate findings across different laboratories. In contrast, early automated algorithms (such as FLOCK) achieve superior accuracy in seconds rather than hours [123].

To address this issue, standard format Gating-ML was proposed, which involves recording gating parameters and logical steps to facilitate result sharing and reproducibility [126]. However, it does not fundamentally overcome the efficiency bottleneck of manual annotation and remains constrained by operator subjectivity.

## 14. Unsupervised Clustering for Cell Type Annotation

With the advancement of computational biology, unsupervised clustering methods such as FlowSOM and PhenoGraph have become mainstream tools for automated cell population identification [127]. These tools algorithmically partition high-dimensional data into distinct cell clusters, which are subsequently annotated on the basis of a known marker expression pattern process known as the “cluster-then-annotate” strategy (as shown in Figure 3b) [128]. For example, Gil-Manso et al. applied FlowSOM to peripheral blood samples from COVID-19 convalescent individuals and healthy controls, generating 15 metaclusters. By examining canonical lineage markers such as CD3, CD4, CD8 and CD19, together with additional differentiation and activation markers, the authors annotated clusters corresponding to conventional lymphocyte subsets including CD4^+^ T cells, CD8^+^ T cells and B cells, as well as distinct memory and activation subpopulations. The complete annotation results, including the cluster-level median fluorescence intensity heatmaps, are provided in the main figures and Supplementary Materials of the study [129]. Unsupervised clustering methods eliminate the need for manual gating to substantially improve efficiency and reduce subjectivity [127].

Recently, several new clustering methods have emerged that offer enhanced scalability and precision. Phenotyping by Accelerated Refined Community-partitioning (PARC) [130], for instance, integrates a hierarchical graph structure and the Leiden algorithm, enabling efficient clustering of large-scale datasets. Similarly, FlowGrid [131] adopts a grid-based density strategy, which improves computational efficiency, particularly for large-scale single-cell RNA sequencing data. In contrast, Specter [132] applies spectral clustering with an ensemble learning approach to enhance clustering accuracy and detect rare populations. Furthermore, Secuer [133] combines spectral clustering with an improved K-nearest neighbor algorithm to significantly reduce computational complexity while providing automatic clustering number estimation. Compared with traditional algorithms, these methods significantly improve the processing of high-dimensional, large-scale data.

Although unsupervised clustering eliminates the need for manual gating and substantially improves efficiency, these technical improvements come with a “reproducibility-bias trade-off.” Specifically, these algorithms are often sensitive to hyperparameter selection, which can lead to unstable results. Furthermore, the annotation phase remains manual: by requiring experts to interpret heatmaps, the subjectivity is simply shifted from the gating plot to the cluster label [108]. Benchmarking these methods is also challenging, as they frequently struggle to distinguish phenotypically similar cell subtypes or may overlook rare populations when the clustering resolution is set too low [24]. These disadvantages limit the utility in studies of complex cellular microenvironments.

## 15. Supervised and Semi-Supervised Methods for Cell Type Annotation

Supervised and semi-supervised learning strategies have been increasingly applied in cell type annotation to improve precision and efficiency. These methods typically leverage partially annotated datasets to learn prior knowledge. It is subsequently generalized to unannotated samples to make them particularly suitable for complex datasets when manual annotation is costly and inconsistent (as shown in Figure 3c). For instance, flowLearn, a semi-supervised method, uses a density-alignment algorithm to transfer gating parameters from expert-annotated prototype samples to new datasets. In a representative application, flowLearn was applied to 2665 additional samples based on a bone marrow reference, achieving accuracy comparable to expert annotations for major hematopoietic populations [122].

Recent advancements in machine learning and deep learning have significantly improved semi-supervised methods for cell type annotation. CyAnno [134] is an integrated framework that combines an ensemble of classifiers with a landmark cell-based representation. CellCNN [135] employs Multiple Instance Learning (MIL) to identify cell subpopulations associated with specific phenotypes. Both methods automate annotation by learning discriminative patterns directly from high-dimensional data, handling sparsity, and eliminating manual gating. Moreover, DeepCyTOF, a representative of the supervised learning method, employs an end-to-end deep neural network architecture to integrate labeled data learning with cross-sample calibration [136]. It achieves a high level of automation when a single manually annotated reference sample and domain adaptation techniques are used to generalize the model to unlabeled datasets.

By combining expert prior knowledge with data-driven models, these approaches substantially enhance the reproducibility of cell type annotation. Critically, the performance of supervised methods is entirely dependent on the quality and representativeness of the training data—a phenomenon known as selection bias. If the reference dataset contains errors or lacks specific rare populations, the model will propagate these inaccuracies to all future analyses [137]. Furthermore, batch effects between the reference and query datasets can severely undermine reproducibility. While domain adaptation techniques (as in DeepCyTOF) attempt to mitigate this, the lack of independent, cross-platform benchmarking datasets makes it difficult to assess how these models perform when faced with the biological variability of real-world clinical samples.

## 16. Fully Automated Methods for Cell Type Annotation

The rapid advancement of deep learning models is accelerating the development of fully automated cell type annotation tools. Recent annotation tools are capable of operating with minimal prior input without labeled training data or only limited marker information. (as shown in Figure 3d) For example, ScType [138] leverages a cross-species marker gene database encompassing 3980 genes across 194 cell types. It utilizes a combined positive/negative marker strategy, demonstrating annotation accuracies of up to 98.6% in complex tissues. Similarly, ScTab [137] applies a nonlinear model architecture based on TabNet, trained on 22.2 million single-cell profiles. It achieved a macro F1 score of 0.83 for cross-tissue annotation, surpassing traditional linear models. While both tools are currently optimized for single-cell transcriptomic data, they are being increasingly adapted for use with CySCP data. These fully automated tools are drastically decreasing the technical barrier for cell-type annotation to efficiently analyze cellular heterogeneity within complex biological systems.

AI-driven data interpretation is increasingly refining cell type annotation in CySCP. Large language models (LLMs) can now decode complex relationships among protein markers, cellular states, and functional phenotypes by incorporating multimodal data, including data from the literature and structured biological databases. This enhances the accuracy of annotating rare or novel cell subsets that may lack well-defined marker panels [139]. Concurrently, graph-based models, such as graph neural networks, excel at modeling intricate protein co-expression networks and hierarchical cellular relationships revealed by high-dimensional flow cytometry data. By learning from high-parameter single-cell protein measurements, these models support annotation while inferring developmental trajectories and cell–cell communication patterns that are not directly observable, providing a systems-level view of cellular behavior and heterogeneity [140]. The synergy of these AI methods with flow cytometry data streamlines the annotation workflow, reduces the reliance on manual gating strategies, and enables a more dynamic interpretation of functional protein expression states in health and disease.

Taken together, no single annotation strategy is universally superior. Manual gating provides the strongest biological interpretability but is limited by poor reproducibility and pronounced operator bias. Unsupervised clustering mitigates subjectivity yet introduces parameter-dependent variability and often exhibits reduced sensitivity for rare cell populations. Supervised and semi-supervised approaches achieve a reasonable balance, but are constrained by training-data bias and limited generalization across datasets. Fully automated methods offer maximal throughput, although their robustness in the context of CySCP remains to be rigorously benchmarked and independently validated. Future investigations should employ standardized benchmark datasets—such as multi-center CyTOF panels with well-defined ground-truth labels—to quantitatively compare the efficiency, reproducibility (such as adjusted Rand index and F1 score), and bias of different methods. These efforts will be critical for developing robust, operator-independent pipelines for reliable cell type annotation in cytometry-based single-cell proteomics.

## 17. Databases for Cytometry-Based SCP

Recently, a growing number of specialized databases have emerged to support experimental design, data storage, and downstream analysis, as shown in Table 3. These databases play a pivotal role in facilitating the analysis of cellular heterogeneity and target identification by promoting standardized data archiving, optimizing antibody panel design, and enabling the development of novel analytical algorithms [141,142].

## 18. Databases for Data Storage and Sharing

As SCP data continues to grow, the need for raw data sharing has become increasingly pressing [153]. To address this problem, several repositories (such as Cytobank [118], FlowRepository [143], SingPro [23], and SPDB [144]) have been developed to curate and organize CySCP data. Specifically, Cytobank [118] supports the management and archiving of flow cytometry, mass cytometry, and other single-cell datasets, provides cloud-based data storage services, and enables seamless interdisciplinary and cross-regional collaboration through web-based platforms. FlowRepository [143], the first open-access repository dedicated to archiving raw flow cytometry data, addresses key challenges in data sharing, such as inaccessibility, incomplete metadata, and inconsistent data processing.

Moreover, SingPro [23] and SPDB [144] are key databases constructed to facilitate data sharing in single-cell proteomics, encompassing not only CySCP data but also MS-based SCP data. These databases provide substantial support for research efforts in the field. SingPro aggregates raw data from 204 studies, encompassing more than 625 million cells and 16,000 proteins across various species, tissues, and disease types. SingPro systematically presents mass cytometry and flow cytometry data alongside detailed experimental metadata and protein expression profiles. Furthermore, the SPDB integrates 143 datasets containing more than 300 million cells and 8000 proteins, covering diverse species, tissues, and disease contexts. Its distinguishing features include standardized preprocessing pipelines, an intuitive user interface, and deep search capabilities from the dataset to the protein level. While both databases offer complementary strengths, SingPro emphasizes comprehensive access to raw data and experimental metadata, allowing for precise data traceability. In contrast, SPDB excels in data integration and analytical versatility, offering powerful tools and broader analytical perspectives for in-depth research.

## 19. Databases for Experiment Design

In the design of CySCP experiments, data quality depends on the ability of the antibody to specifically recognize target proteins. However, the issues associated with antibody products (such as low specificity, batch-to-batch variability, and false positives) [154] severely compromise experimental reproducibility and data reliability. Therefore, antibody accessibility, validation, and applicability should be systematically evaluated in the experimental design phase to ensure scientific reproducibility. Currently, several high-quality antibody databases have been developed to assist in target selection, antibody screening, and multicolor panel design, including Antibodypedia [145], The Human Protein Atlas (HPA) project [146], IMGT/mAb-DB [147], Antibody Registry [148], and ImmPort [149].

Antibodypedia is a public platform that provides antibody information from global suppliers. It covers more than 200,000 antibody entries and includes validation tags along with associated links to the literature for each antibody. In addition, HPA integrates antibody data with protein expression profiles (immunohistochemical staining, subcellular localization, and single-cell RNA expression) at the tissue and single-cell levels. Moreover, IMGT/mAb-DB is a specialized sub-database of IMGT dedicated to structural and functional monoclonal antibody information. It provides high-quality data on heavy and light chain V(D)J gene sequences, antigen-binding regions, and affinity metrics at the molecular level, which serve as a valuable reference for designing recombinant antibodies for multiplex staining or customizing metal-conjugated antibodies in mass cytometry. Moreover, Antibody Registry standardizes antibody usage across platforms and publications by assigning each antibody a unique Research Resource Identifier (RRID) and aids in antibody selection and reproducibility assessment. Furthermore, ImmPort is an immunological data-sharing platform that aggregates antibody panel usage records from major projects, including clone IDs, sources, concentrations, and application contexts.

## 20. Databases for Cell Type Annotation

Cell type annotation typically relies on two types of prior knowledge: gene markers and reference data. Gene markers refer to genes that are highly expressed in specific cell types and serve as critical references for manual gating, clustering-based annotation, or automated annotation methods. Reference data, on the other hand, consist of known data matrices and cell identity labels from previously characterized datasets, which are used as the core for model learning in semi-automated or fully automated annotation processes. In this context, CellMarker [150,151] and CellSTAR [152] databases provide marker-based prior knowledge for identifying key protein markers in cell type annotation. While originally developed for single-cell transcriptomics, they offer valuable reference information for cell type annotation in SCP as well. This curated marker knowledge in these databases is helpful for quickly identifying key protein markers and facilitating a better understanding of cell functions and states.

CellMarker database [150] provides 13,605 markers for 467 human cell types across 158 tissues/sub-tissues, and 9148 markers for 389 mouse cell types across 81 tissues/sub-tissues. Known cell markers are essential for distinguishing different cell populations in single-cell datasets and for supporting downstream analyses. However, reliance on manual curation can lead to delays in updating, making CellMarker less responsive to emerging research hotspots. To address this issue, CellMarker 2.0 [151] was released, providing 36,300 new tissue-cell type-marker entries, covering 474 tissues, 1901 cell types, and 4566 markers. CellMarker 2.0 introduces six web-based analytical tools: cell type annotation, cell clustering, malignancy analysis, differentiation analysis, feature analysis, and communication analysis. Moreover, CellSTAR [152] integrates expression profiles and annotation references from 515 projects and 1679 batches, encompassing 889 distinct cell types across 139 tissues and 18 species. Uniquely, CellSTAR provides expert-curated reference data for annotating hundreds of cell types and integrates tens of thousands of markers to facilitate comprehensive annotation by combining reference profiles with markers.

However, the reference data in CellSTAR are derived from single-cell transcriptomic datasets. While these reference datasets can be utilized for CySCP-based cell type annotation, the inherent discrepancies between transcriptomic and proteomic profiles may lead to reduced accuracy in annotation compared with the use of proteomic data as references. Currently, CySCP reference data are dispersed across various repositories, such as FlowRepository and other repositories previously mentioned, which house relevant datasets for cell type annotation. The integration of these disparate data sources remains a significant challenge in ensuring the consistency and accuracy of annotations within the single-cell proteomics field.

## 21. Conclusions

### 21.1. Current Challenges

Cytometry-based single-cell proteomics is emerging as an indispensable tool because it offers unprecedented resolution and sensitivity in studies of cellular heterogeneity, phenotype profiling, biomarker discovery, and functional monitoring. This review offers detailed descriptions of CySCP, including quantification technologies, analysis pipelines, annotation strategies, and resource platforms. Specifically, this review (1) discusses the strengths and limitations of detection platforms, (2) elucidates comprehensive data processing steps and methods, (3) delineates various strategies for cell type annotation, and (4) summarizes SCP database resources. By integrating these key aspects, this review not only outlines the current technical and analytical landscape of CySCP but also highlights its potential to overcome major challenges in sensitivity, standardization, and data interpretation.

Despite significant progress in the field, several technical challenges continue to impede the broader application and reliability of CySCP. A major issue is the sparsity of high-dimensional CySCP data, where missing or incomplete parameters can limit the accuracy and robustness of analyses. Additionally, signal drift and variations in instrumentation performance across different laboratories can introduce inconsistencies in results, undermining data reliability and reproducibility. Cross-lab reproducibility is another pressing challenge, as differences in protocols, reagents, and instrumentation can lead to substantial variation in experimental outcomes and complicate the integration and comparison of results across diverse studies. Finally, the high operational costs of CySCP technologies, including the costs of instruments, reagents, and data analysis infrastructure, remain a significant barrier to the widespread adoption of the technology, especially in resource-limited settings. These factors also pose substantial challenges to the clinical translation of CySCP, where achieving high reproducibility, cost-efficiency, and standardized workflows is essential for transforming this powerful research tool into a reliable platform for precision medicine.

### 21.2. Future Perspectives

To address the challenges of robustness and reproducibility in CySCP studies, the establishment of standardized and harmonized experimental protocols is paramount. Such standardization should encompass every stage of the analytical pipeline, including uniform procedures for sample preparation, antibody validation, instrument calibration, and long-term performance tracking, thereby minimizing technical variability both within and across laboratories. Equally important is the incorporation of rigorous quality control frameworks, such as the use of internal controls and reference standards, to continuously monitor system performance and ensure data comparability over time. Implementing these measures not only enhances experimental reproducibility and analytical robustness but also represents a critical step toward clinical translation, where reproducible and validated workflows are prerequisites for regulatory compliance and diagnostic reliability [155].

In the clinical context, standardized CySCP pipelines can facilitate multi-center data integration, inter-laboratory benchmarking, and quality-assured proteomic profiling to ultimately reduce variability, operational cost, and technical barriers to adoption in translational and diagnostic settings. These efforts can further build upon existing community-driven initiatives such as MIFlowCyt [156], which defines the minimum information standards for flow cytometry experiments, and the Human Proteome Project [157], which promotes global standardization and data sharing in proteomics. Moreover, tools such as *ANPELA* [158], which facilitate systematic benchmarking and automated workflow selection, are emerging. *ANPELA* can assist researchers in identifying data processing workflows that perform well for a given dataset, thus enhancing the robustness and reliability of the overall data processing procedure.

Exploration of cutting-edge computational strategies and novel model architectures is needed to meet the increasing complexity of biological data. For example, graph neural networks for cell type annotation and deep learning models for correcting batch effects and signal noise should be integrated. Moreover, more refined databases tailored to SCP management, such as reference data repositories specifically designed for SCP cell type annotation, as well as platforms for standardized protein expression profiling and cross-study data integration, should be established to assist with data analysis and storage.

Beyond the development of new computational methods, their practical adoption is critical for improving CySCP data processing and data interpretation. Although many recently proposed algorithms are strong in sensitivity, robustness, or scalability, researchers often rely on familiar pipelines, reflecting a degree of methodological inertia. Encouraging users to evaluate and incorporate newer approaches can help fully leverage advances in the field. In addition, comparing results across multiple preprocessing and analytical strategies, rather than depending on a single workflow, may provide a more reliable assessment of technical and biological variation. These efforts will collectively strengthen the transparency, reproducibility, and analytical rigor of CySCP research.

The convergence of AI and multimodal analysis is expected to shift research from traditional, expert-driven methodologies to more efficient, reproducible, and scalable data-driven precision analytics paradigms. Recently, foundational models in single-cell biology, such as scGPT [159] and scFoundation [160], have emerged. These models rely on the extensive biological principles learned during pretraining on vast single-cell transcriptomic datasets and have shown impressive performance across various downstream tasks, including cell type annotation. However, current foundational model training and evaluation methods focus primarily on single-cell transcriptomics. The development of a novel and robust foundational model for SCP, or even a multimodal model encompassing transcriptomics and spatial modalities, could revolutionize the SCP field, offering tremendous benefits for accurate annotation and data interpretation.

In the future, CySCP-derived protein expression profiles can be aligned with transcriptomic and epigenomic data at the single-cell level to allow cross-modal representation learning. Furthermore, coupling CySCP with spatial proteomics and imaging-based platforms can contextualize protein-level heterogeneity within tissue microenvironments to reveal how cell–cell interactions and spatial context shape functional phenotypes. Collectively, these multimodal and spatially resolved datasets provide the foundation for enhancing the precision of cell annotation, pathway inference, and disease mechanism exploration.

## Figures and Tables

**Table 1:** Comprehensive comparisons of single-cell proteomics technologies, including CFC (conventional flow cytometry), SFC (spectral flow cytometry), MC (mass cytometry), and MS (mass spectrometry) across detection principles, all the major preprocessing steps (compensation/unmixing, transformation, normalization, signal cleaning, and doublet removal), dimensionality, throughput, viability, capability, and current bottlenecks. NA: Not Applicable, meaning the step/feature is not required or relevant to the listed technology.

Feature	CFC	SFC	MC	MS
Detection principle	Fluorescence (PMT, peak-based)	Full emission spectrum	Metal isotopes + ICP-TOF-MS	LC-MS/MS
Maximum practical parameters	15–20	40–60	45–55	1000–5000 proteins
Typical study throughput (cells/day)	10^8^	10^7^–10^8^	10^6^	10^2^–10^3^
Cell viability/sorting	Yes	Yes	No	No
Signal overlap correction	Compensation	Spectral unmixing	Minimal spillover	NA
Compensation	flowCore, AutoSpill	Instrument supplier software (e.g., SpectroFlo software v2.2)	CATALYST, CytoSpill	NA
Transformation	Logicle/arcsinh	arcsinh	arcsinh	Log transformation
Normalization and batch correction	gaussNorm/warpSet	CytoNorm	Bead-based normalization/ CytofBatchAdjust	Median normalization/ComBat
Signal cleaning tools	flowAI, flowClean, flowCut	PeacoQC	PeacoQC	Noise filtering
Doublet/debris removal	FSC-A/H, SSC gating	FSC-A/H, SSC gating	Event length, DNA intercalator	NA
PTM/unbiased discovery	Very limited	Limited	Limited	Excellent
Cost/accessibility	Low	Medium	High	Very high
Data dimensionality	Medium	High	High	Very high
Major remaining bottleneck	Severe fluorescence spillover	Fluorochrome availability, unmixing complexity	Low throughput, destructive	Low throughput, higher cost

**Table 2:** Data processing methods for cytometry-based single-cell proteomics data, including compensation, transformation, normalization, batch effect correction, and signal cleaning.

Abbr.	Tool	Lang. (Package/Library)	Description	Ref.
**Compensation**				
ATS	AutoSpill	R v4.4.2 (autospill v0.2.0)	AutoSpill optimizes spillover matrices via single-color controls, robust regression, and iterative refinement, auto-gating debris for precise correction.	[70]
CAT	CATALYST	R v4.4.2 (CATALYST v1.34.1)	CATALYST uses single-stained antibody capture beads to calculate a spillover matrix and applies a solved compensation matrix to correct spillover in cell data.	[58]
CTS	CytoSpill	R v4.4.2 (CytoSpill v0.1.0)	CytoSpill corrects mass spec data via mixture-model noise splitting, isotopic matrices, and constrained spillover optimization without single-dye controls.	[71]
FLC	FlowCore	R v4.4.2 (flowCore v2.22.1)	FlowCore automates compensation matrix calculation via single-stain controls and batch processes fluorescence overflow in flow cytometry.	[72]
MTC	MetaCyto	R v4.4.2 (MetaCyto v1.32.1)	MetaCyto automates cytometry meta-analysis across flow and mass cytometry data, detecting shared cell populations without manual parameter tuning.	[73]
**Transformation**				
ACS	Arcsinh Transformation	R v4.4.2 (flowCore v2.22.1)	Arcsinh Transformation divides raw data by a cofactor, applies asinh to achieve near-linear data around zero and logarithmic scaling at high values.	[74]
ANN	Asinh with Non-negative Value	R v4.4.2 (flowCore v2.22.1)	Asinh with Non-negative Value subtracts an offset, sets negatives to zero, then applies asinh for processed data.	[74]
ARN	Asinh with Randomized Negative Value	R v4.4.2 (flowCore v2.22.1)	Asinh with Randomized Negative Value offsets data, replaces negatives with low-level normal random values, then applies asinh.	[74]
BEP	Biexponential Transformation	R v4.4.2 (flowCore v2.22.1)	Biexponential Transformation scales cytometry data via a biexponential function, preserving low-intensity linear details and expanding high-intensity logarithmic ranges with tunable parameters to optimize multi-peak resolution.	[75]
BOX	Box–Cox Transformation	R v4.4.2 (flowCore v2.22.1)	Box–Cox transform is a power transformation method adjusted by the parameter λ. It is used to deal with skewed distributions and heteroskedasticity of data to bring them closer to normal or symmetric distributions	[76]
FVS	FlowVS	R v4.4.2 (flowVS v1.4.2)	FlowVS stabilizes cytometry data via asinh transform and Bartlett-optimized parameters, uncoupling fluorescence variance-mean correlations.	[77]
HPL	Hyperlog Transformation	R v4.4.2 (flowCore v2.22.1)	HyperLog is a log-like hybrid transform for negative, zero, and positive values, adapting between logarithmic and linear via one parameter for compensated data.	[78]
LIN	Linear Transformation	R v4.4.2 (flowCore v2.22.1)	Linear transformation can apply a linear function to translate and scale raw measurements without changing their distribution shape.	[79]
LNT	Natural Logarithm Transformation	R v4.4.2 (flowCore v2.22.1)	Natural logarithm transformation (log base e) converts intensity values to the log scale, which compresses high values and can help normalize multiplicative noise.	[72]
LOG	Logarithm Transformation	R v4.4.2 (flowCore v2.22.1)	Logarithmic transformation (commonly log10). performs a non-linear log conversion of signals to reduce heteroscedasticity and spread out low-range values, often yielding a more symmetric distribution of intensities.	[80]
LGT	Logicle Transformation	R v4.4.2 (flowCore v2.22.1)	Logicle merges linear and logarithmic scales for flow cytometry, displaying negative values and wide dynamic ranges while avoiding traditional log artefacts.	[81]
QUA	QuadraticTransform	R v4.4.2 (flowCore v2.22.1)	QuadraticTransform applies quadratic scaling (squaring values) to amplify high-value differences for specific fluorescence channels/parameters.	[72]
SCA	ScaleTransform	R v4.4.2 (flowCore v2.22.1)	ScaleTransform subtracts the minimum value of a marker and divides by its range, rescaling that channel’s values to the [0, 1] interval.	[72]
TRU	TruncateTransform	R v4.4.2 (flowCore v2.22.1)	TruncateTransform truncates data below a set threshold to that value via a lower-bound cutoff before analysis.	[72]
**Normalization**				
BEN	Bead-based Normalization	R v4.4.2 (premessa v0.3.4)	Bead-based Normalization normalizes CyTOF data across runs using spiked-in bead standards. Bead events are identified in each sample, and their median signals are used to calculate normalization factors over time.	[82]
GSN	GaussNorm	R v4.4.2 (flowStats v3.30.0)	GaussNorm eliminates variation between technical samples by aligning salient features (landmarks) in the raw data on a per-channel basis.	[83]
WPS	WarpSet	R v4.4.2 (flowStats v3.30.0)	WarpSet normalizes flow cytometry data sets by aligning high-density regions for each channel.	[84]
**Batch Effect Correction**				
BER	BatchEffectRemoval	Python v3.7.4 (batchEffectRemoval2020)	BatchEffectRemoval is a deep learning method based on residual neural networks (ResNet) and maximum mean difference (MMD) that effectively removes batch effects from biological data by matching multivariate distributions.	[85]
CBT	ComBat	R v4.4.2 (sva v3.8.0)/Python v3.7.3 (pycombat v0.20)	ComBat standardizes data and corrects batch effects via Bayesian and empirical Bayesian methods for cross-batch proteomic/epigenomic integration.	[86]
CTB	CytofBatchAdjust	R v4.4.2 (CytofBatchAdjust)	CytofBatchAdjust automatically corrects for batch effects in CyTOF data by introducing technical duplicate samples (anchors) in each batch and calculating tuning parameters for each channel between batches based on percentiles.	[87]
FMN	FastMNN	R v4.4.2 (batchelor v1.27.1)	FastMNN accelerates MNN matching in PCA space, eliminates batch differences via local linear transformations, balancing efficiency and cross-batch integration.	[86]
HMN	Harmony	R v4.4.2 (harmony v1.2.4)/Python v3.7 (harmonypy v0.2.0)	Harmony alternates clustering/models in PCA space, balancing batch removal and heterogeneity to prevent overcorrection.	[86]
SNM	Scanorama	Python v3.8 (scanorama v1.7.4)	Scanorama stitches data via panoramic neighbor mapping and corrects nonlinear batch bias with hyperplane projections for heterogeneous single-cell integration.	[86]
SRC	Seurat v3 CCA	R v4.4.2 (mixOmics v6.32.0)	Seurat v3 CCA builds cross-batch subspaces via CCA and corrects gene matrix batch bias using anchor mapping.	[86]
SRR	Seurat v3 RPCA	R v4.4.2 (Seurat v5.4)	Seurat v3 RPCA replaces CCA with RPCA for batch reduction, handles large-scale batch effects via anchor integration, cutting computational complexity.	[86]
**Signal Cleaning**				
FAI	FlowAI	R v4.4.2 (flowAI v1.40.0)	FlowAI enhances flow cytometry data stability by detecting and removing anomalies from signal mean/variance shifts during acquisition.	[33]
FCL	FlowClean	R v4.4.2 (flowClean v1.48.0)	FlowClean improves flow data by tracking cell shifts and detecting fluorescence anomalies via central log-ratio and variable point analysis.	[88]
FCU	FlowCut	R v4.4.2 (flowCut v1.20.0)	FlowCut enhances flow data by detecting/removing time-chunked anomalies via statistical/Z-score/density thresholds.	[89]

**Table 3:** Databases for cytometry-based single-cell proteomics data.

Database	Website	Description	Ref.
**Data Storage and Sharing**			
Cytobank	http://www.cytobank.org/ (accessed on 15 April 2026)	Cytobank is a platform that allows researchers to annotate, analyze, and share results along with the underlying single-cell data.	[118]
FlowRepository	http://flowrepository.org/ (accessed on 15 April 2026)	FlowRepository hosts flow cytometry datasets linked to publications, enabling MIFlowCyt-compliant upload/annotation/sharing for access/reproducibility.	[143]
SingPro	https://idrblab.org/singpro/ (accessed on 15 April 2026)	SingPro systematically provides the SCP raw data for both mass spectrometry-based and flow cytometry-based studies and explicitly describes experimental detail for SCP study and expression profile of any studied protein.	[23]
SPDB	https://scproteomicsdb.com/ (accessed on 15 April 2026)	SPDB hosts single-cell proteomic data (antibody/mass spectrometry-based), offering search/visualization/download in unified formats.	[144]
**Experiment Design**			
Antibodypedia	https://www.antibodypedia.com/ (accessed on 15 April 2026)	Antibodypedia shares validated human protein antibodies via community-driven scoring, aiding researcher selection.	[145]
Human Protein Atlas (HPA)	https://www.proteinatlas.org/ (accessed on 15 April 2026)	Human Protein Atlas maps single-cell protein expression/distribution via antibody/transcriptomic data, aiding biology/disease research.	[146]
IMGT/mAb-DB	https://www.imgt.org/mAb-DB/ (accessed on 15 April 2026)	IMGT/mAb-DB provides therapeutic mAbs’ sequence/structure/mechanism data via standardized queries/visualization, supporting drug R/D and clinical decisions.	[147]
Antibody Registry	https://antibodyregistry.org (accessed on 15 April 2026)	Antibody Registry assigns RRIDs to track/cite commercial/nonprofit/custom antibodies, enhancing research reproducibility and transparency.	[148]
ImmPort	https://www.immport.org/ (accessed on 15 April 2026)	ImmPort, an NIAID portal, shares clinical/molecular data, promoting standards, discovery, and education.	[149]
**Cell Type Annotation**			
Cellmarker	http://xteam.xbio.top/CellMarker/ (accessed on 15 April 2026)	CellMarker compiles human/mouse cell markers from single-cell/experimental/literature data, providing tissue-specific markers and statistics to support precise identification, functional studies, and biomedical reproducibility.	[150]
Cellmarker2.0	http://bio-bigdata.hrbmu.edu.cn/CellMarker/ (accessed on 15 April 2026)	CellMarker 2.0 expands with 36K entries, 48 sequencing sources, 6 tools (annotation/clustering/malignancy), covering 656 tissues/2578 cell types/26K markers, enabling precise single-cell analysis for immunology/tumor research.	[151]
CellSTAR	https://idrblab.org/cellstar (accessed on 15 April 2026)	CellSTAR provides expert-annotated reference data for first-time annotation of hundreds of cell types, integrating thousands of markers for joint analysis.	[152]

## Data Availability

No new data were created or analyzed in this study. Data sharing is not applicable to this article.
